# Bypassing the Blood-Central Nervous System (CNS) Barrier: A Dual-Axis Framework for Amyotrophic Lateral Sclerosis (ALS) Utilizing Glymphatic-Lymphatic Clearance and In Situ Chaperone Synthesis

**DOI:** 10.7759/cureus.117043

**Published:** 2026-09-28

**Authors:** Huan-Wei Chen

**Affiliations:** 1 Chiropractic, Private Chiropractic Practice, Vancouver, CAN

**Keywords:** active craniospinal tensioning (act), amyotrophic lateral sclerosis (als), blood-cns barrier (bcnsb), cerebral venous preconditioning (cvpc), dural pull-recoil, glymphatic-lymphatic clearance, heat shock factor 1 (hsf-1), sod1 proteinopathy, suboccipital venous occlusion-rebound, tdp-43 proteinopathy

## Abstract

Amyotrophic lateral sclerosis (ALS) is characterized by prion-like propagation of misfolded TAR DNA-binding protein 43 (TDP-43) and superoxide dismutase 1 (SOD1) across motor neuron pools. Because any circulating pharmacological agent must cross the blood-brain and blood-spinal cord barriers (BBB and BSCB), this review proposes active craniospinal tensioning (ACT) as a mechanical, non-pharmacological route to disease modification that acts on cerebrospinal fluid (CSF) and venous pressure dynamics rather than requiring penetration of these barriers. ACT is proposed to engage a single Monro-Kellie/Queckenstedt pressure continuity through two concurrent mechanisms: suboccipital venous occlusion-rebound, hypothesized to transiently raise and release craniospinal CSF pressure via venous compression and thereby accelerate venous-side glymphatic efflux; and dural pull-recoil, hypothesized to generate a reciprocal CSF pressure gradient via spinal elongation, agitating perivascular CSF directly while inducing a coupled venous pressure gradient. Because both mechanisms act concurrently on a shared venous-side target, the combined maneuver is proposed to be more potent than either delivered alone, as in prior work on pelvis-stabilized axial spinal traction (PSAST), which lacks the occlusion-rebound component.

A secondary, hypothesis-generating micro-biochemical axis, cerebral venous preconditioning (CVPC), proposes that the same occlusion-rebound cycle produces capillary/venule wall strain sufficient to activate heat shock factor 1 (HSF-1) across the neurovascular unit, independent of oxygen tension, with a possible but uncharacterized contribution from hypoxia-inducible factor 1-alpha (HIF-1α). The two routes are non-exclusive, with the macro-fluidic axis alone proposed as sufficient. This framework is entirely theoretical, requires preclinical and clinical validation, and is intended as a template for related proteinopathy-driven neurodegenerative diseases, including Parkinson's disease, Huntington's disease, spinocerebellar ataxia, and fatal familial insomnia, addressed in forthcoming work.

## Introduction and background

Amyotrophic lateral sclerosis (ALS) is the most common adult-onset motor neuron disease, characterized by progressive degeneration of upper and lower motor neurons (UMN and LMN) in the motor cortex, brainstem, and spinal cord. Average onset is 58-60 years, with overall survival of only 3-4 years [1]; bulbar-onset disease carries a comparatively worse prognosis, with median survival from symptom onset of approximately 27 months [2]. Patients present with limb-onset weakness or bulbar-onset symptoms, such as dysarthria and dysphagia, and most eventually die of respiratory insufficiency [1].

At the center of ALS pathogenesis is a proteotoxic process defined almost entirely by intracellular misfolding: cytoplasmic mislocalization and aggregation of TAR DNA-binding protein 43 (TDP-43) occur in approximately 97% of cases, sporadic and familial alike, while misfolded superoxide dismutase 1 (SOD1) accounts for a smaller subset, roughly 2%, arising from any of over 200 described mutations [3]. Both proteins behave in a prion-like manner, templating conformational change in native protein and propagating a pathological state cell-to-cell along the neuroaxis rather than remaining confined to the neuron of origin, consistent with the clinical observation that ALS symptoms typically begin focally and spread contiguously [4]. ALS and frontotemporal dementia (FTD) are increasingly recognized as points on a shared clinico-genetic spectrum, with overlapping TDP-43 pathology [4]; the macro-fluidic mechanism proposed here for interrupting TDP-43 propagation may therefore extend to FTD without requiring a separate disease-specific framework. This templated propagation is the process this framework's primary intervention targets: any strategy capable of physically removing suspended misfolded protein from the extracellular space before it templates a neighboring cell has a plausible route to slowing disease spread, independent of the target cell's own biochemical stress-response capacity.

This proteotoxic profile is part of why ALS was chosen as the disease-specific context for developing this framework's staged clinical validation pathway, though not the only reason. Compared with diseases such as Parkinson's and Alzheimer's, where multiple co-occurring misfolded species (alpha-synuclein, amyloid-beta, tau) commonly contribute to pathology, ALS proteinopathy is described predominantly through a single dominant species, TDP-43, present in approximately 97% of cases, with SOD1 accounting for a separate, genetically defined minority. This offers a cleaner single-mechanism test case than these diseases for a framework whose primary claim rests on extracellular clearance of a specific misfolded protein, though it is not unique in this respect: monogenic conditions such as Huntington's disease and fatal familial insomnia offer comparably clean, arguably cleaner, single-protein profiles. ALS was ultimately selected for practical reasons: a substantially larger patient population than that of these rarer monogenic diseases, and a short, predictable disease course, typically fatal within 3-4 years, that permits a faster and more clearly interpretable readout within a staged validation timeline.

A second, longer-established pathogenic thread runs alongside protein misfolding: glutamate excitotoxicity. Astrocytic excitatory amino acid transporter 2 (EAAT2) in humans, also known as glutamate transporter 1 (GLT-1) in rodents, is substantially reduced in both sporadic ALS patients [5,6] and SOD1 mouse models [7], predominantly via translational or post-translational loss, including oxidative/nitrosative inactivation [7] and caspase-3-mediated cleavage of its C-terminus [8]. This underlies riluzole's pharmacological basis [9] and establishes astrocytic transporter integrity as a determinant of motor neuron survival independent of any protein-aggregation pathway [10]. This pathway is addressed later as a secondary consideration; the primary intervention here targets proteotoxic propagation directly.

Current pharmacological options only modestly slow ALS progression: riluzole extends survival by 2-3 months [11], while edaravone has been shown to slow functional decline over 24 weeks in selected patients, without an established survival benefit [12]. Tofersen, an antisense oligonucleotide (ASO) approved for SOD1-mutant ALS, illustrates the barrier constraint facing central nervous system (CNS)-targeted biologics directly: because ASOs cannot cross the blood-brain barrier (BBB), tofersen must be administered by repeated intrathecal injection, bypassing rather than crossing the barrier [13]. This restriction extends to the spinal cord via its own blood-spinal cord barrier (BSCB), which is structurally more permeable than the BBB at baseline and is disrupted early in disease; human cervical cord tissue from ALS patients shows direct pericyte loss and vascular leakage [14]. The BBB and BSCB are collectively termed the blood-CNS barrier (BCNSB) in motor neuron disease, reflecting the need for any prospective non-invasive intervention to address both compartments simultaneously [15].

The glymphatic system offers a physiological precedent for this kind of barrier-independent clearance, and carries a structural asymmetry directly relevant to this framework. Perivascular CSF influx into the brain parenchyma is driven predominantly by arterial pulsation, whereas the corresponding perivenous efflux of interstitial waste is comparatively passive, lacking a dedicated pulsatile driver, before this fluid is carried onward toward the meningeal lymphatics [16,17]. This leaves the venous-side perivascular interface, immediately adjacent to astrocytic endfeet, structurally prone to slower, less actively agitated clearance than the arterial-side pathway enjoys. Direct evidence of glymphatic involvement in ALS specifically is recent but growing: diffusion tensor image analysis along the perivascular space (DTI-ALPS), a non-invasive proxy for glymphatic function [18], has shown reduced indices in ALS patients compared with controls in several studies since 2024 [19,20], including a longitudinal cohort reporting progressive decline relative to a related motor neuron disorder [19]. This evidence remains early and is itself cerebral rather than spinal; the whole-CNS mechanism proposed here therefore extrapolates from cranial glymphatic biology to spinal cord anatomy, via the continuity of the craniospinal CSF compartment, rather than resting on directly demonstrated spinal findings.

The paragraphs above establish ALS pathophysiology as currently understood in the field; the remainder of this Introduction turns from established disease biology to the physiological rationale this framework proposes to build, and readers should treat this as the point where established findings give way to this paper's own hypothesis.

Separately, stress-survival pathways such as remote ischemic preconditioning (RIPC) and local cerebral ischemic preconditioning (LIPreC) are well established outside the CNS and, to a limited degree, within it. Both act via arterial occlusion, producing true ischemia in which induction of heat shock factor 1 (HSF-1) and hypoxia-inducible factor 1-alpha (HIF-1α) is coupled through a shared hypoxic/metabolic trigger, typically sustained for minutes to tens of minutes [21,22]. Cerebral venous preconditioning (CVPC) is mechanistically distinct from, rather than a faster or more local variant of, ischemia: it occludes venous outflow while arterial inflow continues, producing a congested rather than ischemic bed. Mechanical wall strain from this congestion is proposed to activate HSF-1 independently of oxygen tension [23,24]; separately and non-exclusively, the same trapped pool may also provide a hypoxic stimulus that stabilizes HIF-1α. However, HIF-1α's induction kinetics under sub-2-minute exposure remain uncharacterized [25], leaving this contribution open rather than established. This proposed decoupling from a shared hypoxic/metabolic trigger distinguishes CVPC from ischemic preconditioning, regardless of whether the HIF-1α component materializes. The central gap this framework addresses remains the venous-side clearance asymmetry of the macro-fluidic axis.

Active craniospinal tensioning (ACT), introduced in our prior work [26,27], builds on pelvis-stabilized axial spinal traction (PSAST) [28,29]. Active craniospinal tensioning, self-administered (ACT-SA) [26,27], delivers this mechanism via a participant-driven squat. Most recently, a variant, active craniospinal tensioning, operator-driven (ACT-OD) [30], was introduced to deliver the same mechanism via a practitioner-operator instead, extending access for patients who cannot generate a voluntary squat. This extension is directly motivated by ALS's clinical course: as UMN and LMN involvement progresses, patients who could initially self-administer ACT-SA may lose the motor capacity to do so, making operator-driven delivery necessary to sustain treatment access throughout the disease trajectory rather than only in the early stages. ACT-SA and ACT-OD are accordingly treated as mechanistically equivalent instances of a single technique, differing only in force source (participant- versus operator-generated) for the same upright combination of dural pull-recoil and suboccipital venous occlusion-rebound [30], together with its proposed biochemical correlate CVPC [23,27]. For ambulatory patients, either mode is viable, with ACT-SA being the more convenient default; for non-ambulatory patients, ACT-OD is the only option. Together, the two modes extend ACT's reach across the full range of ambulatory capacity encountered over the ALS disease course.

The magnitude of dural pull-recoil depends on the spine's ability to elongate under load, which may be reduced, independent of motor status, by kyphoscoliosis, spinal fusion, ankylosing changes, osteoporotic vertebral collapse, or severe contracture, and is difficult to correct with operator-driven force. The favorable spinal elongation achieved in the foundational case report [28] should accordingly not be assumed representative of the ALS population generally. Suboccipital venous occlusion, by contrast, targets a discrete, superficial anatomical structure that is largely independent of overall spinal flexibility and is expected to be deliverable in nearly all patients, regardless of spinal anatomic status. This independence is assumed rather than directly demonstrated: whether a rigid or fused spinal column alters how applied tension distributes through the strap-neck interface during ACT-OD lift-off, potentially attenuating the force actually delivered to the suboccipital plexus, has not been characterized.

In this article, we extend that proposal into a formal framework for ALS specifically, organized around a primary and a secondary axis. The macro-fluidic axis rests on a single physical basis applied in two directions: the Monro-Kellie doctrine, which holds that CSF, cerebral blood, and brain parenchyma occupy a fixed-volume compartment such that a change in one must be offset by a reciprocal change in another, and the Queckenstedt test, which demonstrates this coupling directly by showing that external jugular venous compression raises CSF pressure. ACT is proposed to engage the same continuity at both ends. Suboccipital venous occlusion-rebound is hypothesized to apply the coupling in the Queckenstedt test's original direction, using transient venous compression to raise and then release craniospinal CSF pressure, thereby directly accelerating venous-side glymphatic efflux. Dural pull-recoil is proposed to apply the same coupling in reverse, a reverse Queckenstedt effect in which elongation and release of the spinal dural sac is hypothesized to initiate the CSF-side perturbation rather than the venous side, and is proposed to act on glymphatic clearance through both the CSF compartment itself and a coupled venous pressure gradient. Because the two mechanisms are delivered concurrently within the same maneuver and converge on a shared venous-side target, the combined cycle is proposed to be more potent than either delivered alone, though whether this convergence yields additive or synergistic effects, rather than redundant engagement of the same clearance pathway, has not been established.

The secondary micro-biochemical axis, CVPC, considers whether the same occlusion-rebound cycle also activates HSF-1 across the neurovascular unit, with hypoxic HIF-1α stabilization left open rather than excluded. It is termed secondary in a structural sense, targeting a downstream consequence of proteotoxic stress rather than the propagation step this framework's core claim depends on, not as a claim about its relative plausibility.

## Review

Literature search methodology

This is a narrative, hypothesis-generating review rather than a systematic review, reflecting the interdisciplinary scope of a mechanistic framework spanning CSF/venous physiology, glymphatic-lymphatic clearance, and ALS-specific proteinopathy; no PRISMA-style screening flow diagram was generated, consistent with this format, and the process below is reported for transparency rather than as a claim of systematic completeness.

Literature was identified through PubMed/MEDLINE as the primary database, supplemented by manual reference-list screening (snowballing) from key mechanistic sources, and targeted retrieval of foundational physiology papers predating consistent keyword indexing. Searches were conducted through September 2026. No lower date bound was applied, reflecting the deliberate inclusion of foundational physiology literature (e.g., Monro-Kellie/Queckenstedt-era sources from as early as the twentieth century) alongside recent glymphatic and ALS-specific findings concentrated in 2023-2025; this dual-era approach was necessary because the framework rests on long-established physiological principles applied to recently characterized disease biology, and restricting the search to a recent window would have excluded the mechanistic foundation the framework depends on.

Search terms combined, using AND/OR Boolean operators, across six queries: ("amyotrophic lateral sclerosis" OR ALS) AND ("glymphatic" OR "perivascular clearance" OR "DTI-ALPS"); ("Monro-Kellie" OR "Queckenstedt") AND ("cerebrospinal fluid pressure" OR "venous pressure"); ("heat shock factor 1" OR "HSF-1") AND ("ischemic preconditioning" OR "venous occlusion" OR mechanotransduction); ("TDP-43" OR SOD1) AND ("prion-like" OR propagation); ("blood-brain barrier" OR "blood-spinal cord barrier") AND (ALS OR "antisense oligonucleotide"); (arimoclomol OR ceftriaxone) AND ALS AND trial.

These six queries returned 40, 26, 8, 392, 521, and 40 records, respectively, for a combined total of 1,027 records before deduplication and screening. Off-topic cell biology or non-neurological literature unrelated to CNS mechanotransduction was excluded at the title/abstract level. After deduplication, title/abstract screening against the inclusion and exclusion criteria below, and supplementation via reference-list snowballing for foundational physiology sources predating consistent keyword indexing, 50 references met the criteria for citation and are referenced throughout this review [1-50]. No quantitative evidence synthesis, meta-analysis, or meta-regression was planned or performed; the reviewed literature is synthesized narratively throughout, consistent with this manuscript's hypothesis-generating rather than systematic design.

Inclusion criteria included peer-reviewed, English-language original research articles, randomized controlled trials, and review articles addressing (a) ALS epidemiology, pathophysiology, or pharmacotherapy; (b) CSF, venous, or glymphatic-lymphatic fluid dynamics; (c) mechanotransductive or hypoxic HSF-1/HIF-1α induction; (d) prior ACT/PSAST technical reports by the author, included as foundational prior work rather than independent literature. Exclusion criteria included non-peer-reviewed sources, unreviewed preprints, non-English-language articles, conference abstracts without full-text availability, and sources without direct mechanistic relevance to either the macro-fluidic or micro-biochemical axis. A single reviewer (the author) conducted title/abstract screening and reference-list snowballing, without a second independent screener or a formal risk-of-bias or quality-appraisal tool, consistent with this manuscript's status as a narrative rather than a systematic review.

No primary studies of ACT or CVPC as described in this framework exist in the literature; this absence is itself consistent with, and explicitly acknowledged as, this framework's theoretical status, which is addressed further in the "Limitations" section.

The macro-fluidic axis

Mechanical Clearance as a Primary Route to Blocking Prion-Like Propagation

ALS pathology, as established above, spreads substantially through templated, cell-to-cell propagation of misfolded TDP-43 and SOD1 [3,4]. This gives mechanical clearance a disease-modifying rationale distinct from and independent of any biochemical cytoprotection: removing suspended misfolded protein from the extracellular and perivascular spaces before it can template a neighboring cell, thereby directly interrupting propagation, regardless of the recipient cell's biochemical state. This section develops the physical basis for that claim. This mechanism depends on the maneuver's force actually crossing the physiological thresholds required to occlude the suboccipital venous plexus and fully tension the dural sac. At the 60%-70% body-weight parameters established [30], ACT is proposed to cross both thresholds reliably, though the applied load parameter itself has not been confirmed to produce the intended physiological occlusion and tensioning effect, making the proposed CSF-to-venous coupling a physically plausible event rather than a nominal one.

Suboccipital Venous Occlusion-Rebound: The Monro-Kellie/Queckenstedt Mechanism in Its Original Direction

Of the two components comprising the macro-fluidic axis, this mechanism rests on the most directly established physiology. The Monro-Kellie doctrine holds that CSF, cerebral blood, and brain parenchyma occupy a fixed-volume cranial compartment, such that a reciprocal change in one offsets displacement of another; modern phase-contrast magnetic resonance imaging (MRI) confirms this coupling directly, with jugular venous compression shown to alter cerebral CSF flow dynamics and brain tissue stiffness in healthy volunteers [31]. The Queckenstedt test demonstrates the same coupling diagnostically, using bilateral jugular compression to raise CSF pressure by obstructing venous outflow and assess spinal canal patency, and continues to be used as a research tool despite its decline as a routine diagnostic test [32], recently repurposed to derive quantitative descriptors of the CSF pulsatility curve rather than solely to test spinal canal patency [33]. Suboccipital venous occlusion-rebound applies this same coupling in the Queckenstedt test's original direction, substituting a suboccipital compression for the test's diagnostic jugular compression, applied to the plexus that dominates cranial venous outflow in erect posture [34,35] rather than to the jugular veins directly, exploiting the same principle that impeding venous outflow reliably and measurably raises venous pressure upstream of the occlusion.

The intracranial dural sinuses and the suboccipital venous plexus, as defined in our prior work [27] to include the vertebral venous plexus and posterior emissary channels, form a single, continuous, valveless network with documented direct anastomoses at the craniocervical junction [36,37]. A localized suboccipital occlusion is therefore proposed to produce a sustained elevation of pressure not merely at the occlusion site but throughout the intracranial compartment, while arterial inflow continues. This distinguishes the occlusion phase's reach from the spinal cord's: thoracic, lumbar, and sacral cord segments drain through their own segmental veins into the azygos, hemiazygos, and lumbar venous systems, bypassing the craniocervical bottleneck entirely, so suboccipital occlusion is not expected to elevate venous pressure in the spinal cord directly. Upon release, the accumulated intracranial pressure discharges as a surge through the same network. Whether this surge produces a comparably prominent hemodynamic effect at the thoracic, lumbar, and sacral levels, rather than a minor or negligible one, has not been characterized and is not assumed in either direction here. Even so, the anatomical continuity of the venous network makes some secondary rebound-phase extension into the spinal venous drainage plausible, although the occlusion phase itself cannot reach the spinal cord.

Because this mechanism relies on deliberately provoking transient venous congestion, it raises a safety consideration distinct from ordinary mechanical loading: rapid venous congestion sufficient to approach a presyncope threshold involves a transient elevation of intracranial and venous pressure and a reduction in cerebral perfusion pressure. In a population such as ALS, where autonomic involvement, cardiovascular deconditioning, and fall risk from progressive weakness may all be present, this warrants supervised application, monitoring for excessive or prolonged presyncope, and caution in patients with known cardiovascular instability, syncope history, or inability to communicate symptoms during the maneuver, pending the instrumented safety data called for in the "Methodological framework and priorities for clinical validation" section.

Dural Pull-Recoil: The Same Mechanism in Reverse

ACT's second component applies the same Monro-Kellie/Queckenstedt coupling in reverse, generating two distinct effects from axial elongation and release of the spinal dural sac, which initiates a CSF-side pressure gradient. First, and most directly, the perivascular and interstitial spaces where glymphatic exchange occurs are themselves CSF-filled compartments, so this pressure gradient acts directly on this fluid, agitating and suspending adherent waste throughout the perivascular and interstitial CSF space within the same medium the gradient itself displaces [27]. Second, this same CSF pressure gradient reciprocally induces a corresponding venous pressure gradient wherever it reaches a given region of brain parenchyma, through the same fixed-volume Monro-Kellie coupling that links the craniospinal compartments; it is this coupled venous pressure gradient, acting on the same perivascular and interstitial CSF space from the venous side, that connects dural pull-recoil to the same venous-side glymphatic target addressed directly by suboccipital venous occlusion-rebound above. This is the reverse Queckenstedt effect: the CSF compartment, rather than the venous compartment, is the initiating perturbation. These two effects, direct CSF-side agitation and coupled venous-side agitation, are complementary rather than competing: the direct is intra-compartmental, acting within the CSF itself [27]; the coupled is cross-compartmental, reaching the venous side via the Monro-Kellie doctrine.

The direct pathway depends solely on the presence of CSF-filled perivascular and interstitial spaces, a structural feature present along the full length of the spinal cord [38,39] and brain [16,17]; accordingly, it is proposed to act throughout the craniospinal axis, including the thoracolumbar cord. The coupled pathway has a narrower proposed reach, plausibly attenuated by the valveless, highly compliant epidural venous plexus running the length of the spinal canal, though this has not been directly measured and is not assumed here. Cranially, by contrast, the closed, non-distensible cranial vault is the physical basis of the Monro-Kellie relationship itself, so this pathway is proposed to reach the entire intracranial compartment nearly uniformly through this same coupling.

Mechanical and vascular approaches to enhancing glymphatic clearance are not unprecedented: focused ultrasound with circulating microbubbles mechanically enhances glymphatic transport via acoustic cavitation on the perivascular wall [40], and oscillatory vasomotion, whether norepinephrine-driven during sleep or sensory-evoked, accelerates glymphatic clearance through vascular oscillation [41,42]. These findings support the general plausibility that mechanical or vascular perturbation can drive glymphatic clearance; whether ACT produces this effect as described has not been directly measured, and the model remains an untested, falsifiable hypothesis.

Cranially, where suboccipital venous occlusion-rebound's whole-brain reach is well grounded in established venous-compression physiology, pull-recoil's role is proposed to be primarily agitatory, dislodging adherent protein throughout the perivascular and interstitial CSF space, thereby placing it in suspension for removal by the venous occlusion-rebound mechanism above. In the spinal cord, where occlusion-rebound's reach is limited to a secondary, uncharacterized rebound effect (see "Suboccipital Venous Occlusion-Rebound"), the direct CSF-side pathway of dural pull-recoil is proposed as the primary mechanism of both agitation and removal, rather than agitation alone.

The micro-biochemical axis

A Secondary, Mechanotransductive and Hypoxic Route to In Situ Chaperone Synthesis

Suboccipital venous occlusion-rebound is proposed as a single physical event with two coupled consequences: the macro-fluidic drainage-acceleration effect developed above, and a separate, localized biochemical stress response, corresponding to the in situ chaperone synthesis referenced in this framework's title and termed CVPC [27]. This axis remains secondary for the reason established in the Introduction: it targets a downstream cellular consequence rather than the propagation step itself.

A Dual-Route Trigger: Mechanical Strain and Hypoxia

The companion Alzheimer's disease framework proposed mechanical strain alone as a sufficient trigger for HSF-1 activation [23,24]; this ALS framework extends that model to include a second, independent hypoxic route, offering redundancy rather than resting on a single unproven pathway. Strain-induced signaling via membrane tension and channel gating is understood to initiate more rapidly than the transcriptional/translational heat shock protein 70 (HSP70) response it ultimately produces, though the precise onset kinetics under ACT's specific loading conditions have not been characterized [24]. Separately, suboccipital venous occlusion creates a deoxygenating venous pool distinct from arterial ischemia; HIF-1α induction kinetics during sub-2-minute exposure remain uncharacterized, though the foundational kinetic study's shortest tested exposure already showed detectable induction, establishing an upper bound of 2 minutes for onset but not a lower bound [25], leaving a hypoxic contribution open rather than excluded. This reasoning applies with equal force to the companion Alzheimer's framework [23]. These two routes are proposed as plausible and non-exclusive: CVPC does not require both to succeed, and either occurring independently would be sufficient to activate HSF-1 and/or HIF-1α across the neurovascular unit.

Candidate Site of Action: The Neurovascular Unit

The candidate site of action is the neurovascular unit, comprising capillary and venule endothelium, pericytes, and astrocytes, positioned to bear mechanical load from venous-side wall distension. Mechanical stress alone is established to activate HSF-1 and induce HSP70 in vascular tissue independent of ischemia, demonstrated both in cultured vascular smooth muscle [24] and in vivo in rat aorta subjected to elevated blood pressure [43], and is proposed here to extend to the venous side of the cerebral neurovascular unit by the same physical principle, though this specific extension, including which of these cell types would respond and by what transducer, has not been directly tested. No specific molecular transducer for this response is proposed or assumed at this site; the claim rests on the general precedent that mechanical/hemodynamic stress activates HSF-1 in vascular tissue, not on a demonstrated pathway specific to cerebral endothelium, pericytes, or astrocytes.

If HSF-1/HSP70 induction occurs as proposed in any of these cell types, a plausible downstream consequence is stabilization of astrocytic EAAT2 against post-translational degradation, protecting motor neurons indirectly via reduced glutamate excitotoxicity without requiring the motor neuron's own HSF-1 machinery, which is documented to have a high activation threshold and to fail to activate HSF-1 under the same conditions that reliably induce it in other cell types [44]. This contrast is the primary reason a non-neuronal source of HSF-1/HSP70 induction is of interest in ALS specifically: if motor neurons cannot mount this response themselves, protection would have to arise from a paracrine or non-cell-autonomous source, of which astrocytic EAAT2 stabilization is one candidate route among others; direct paracrine chaperone transfer to motor neurons remains a possible but lower-priority alternative, and neither pathway is required by the framework's primary macro-fluidic claim.

Precedent and Contrast: Prior Pharmacological Trials

Two pharmacological trials targeting overlapping downstream biology, ceftriaxone (EAAT2 upregulation) [45] and arimoclomol (HSF-1 co-induction) [46], did not improve outcomes in unselected ALS. However, a smaller SOD1-restricted arimoclomol trial showed a statistically inconclusive but favorable trend [47], plausibly reflecting a distinct carboxyl terminus of Hsc70-interacting protein (CHIP)-mediated route [48]. Unlike tofersen, which is delivered intrathecally because it cannot cross the BBB, both ceftriaxone (intravenous) and arimoclomol (oral) were shown in their respective trials to achieve therapeutic CSF concentrations [45,46], so their failure cannot be attributed to the barrier-exclusion problem. CNS penetration was achieved in both cases, yet neither trial demonstrated the downstream biological effects (EAAT2 upregulation and sustained HSF-1 activation) required for efficacy. This points to a target-engagement or pathway-responsiveness limitation within CNS tissue itself, not a delivery failure, and it is this distinction, not a shared barrier problem, that CVPC's local, non-pharmacological, mechanotransductive delivery is proposed to test. Whether a physiological trigger can activate HSF-1 more effectively than pharmacological co-induction in a cell population with a documented activation deficit [44] is an open, untested question, not assumed in CVPC's favor. 

The framework's primary mechanistic claim rests on the macro-fluidic axis developed above, which acts directly on prion-like propagation and requires no target-cell stress response (Table 1). Table 1's mechanistic descriptions are stated in abbreviated form for tabular concision; unless a citation is given, all descriptions of ACT's or CVPC's physical action, target, or reach should be read as hypothesized rather than established, consistent with the hedging convention used throughout this review.

**Table 1:** Proposed mechanisms, anatomical reach, and evidentiary status "Primary" and "secondary" designate each axis's proposed relationship to ALS's core propagation pathology (the macro-fluidic axis acts directly on propagation; the micro-biochemical axis targets a downstream cellular consequence), not relative confidence or evidentiary support. Both axes are hypothesis-generating and untested. Dural pull-recoil and suboccipital venous occlusion-rebound are delivered as two concurrent, mechanistically distinct effects of a single ACT maneuver rather than as sequential or separable techniques, sharing identical timing and loading parameters: concurrent onset, a ~5-second hold at the 60%-70% body-weight parameters established in Chen [30] (ACT-OD documented at ~68% body weight), and concurrent rebound/recoil on release, operationally realized in ACT-OD as full lift-off from the seated position. This shared timing is hypothesized and has not been directly measured. Within the macro-fluidic axis, venous occlusion-rebound is treated as the more evidence-secure component, given its grounding in established venous-compression physiology; dural pull-recoil's two pathways are treated as plausible but less directly evidenced, particularly regarding the actual reach of Monro-Kellie coupling under ACT's specific conditions, pending direct measurement. ACT-OD: active craniospinal tensioning, operator-driven; CHIP: carboxyl terminus of Hsc70-interacting protein; CSF: cerebrospinal fluid; CVPC: cerebral venous preconditioning; EAAT2/GLT-1: excitatory amino acid transporter 2/glutamate transporter 1; HIF-1α: hypoxia-inducible factor 1-alpha; HSF-1: heat shock factor 1; HSP70: heat shock protein 70; LMN: lower motor neuron; SOD1: superoxide dismutase 1; TDP-43: TAR DNA-binding protein 43; UMN: upper motor neuron.

	Suboccipital venous occlusion-rebound	Dural pull-recoil	CVPC (secondary, hypothesis-generating)
Axis	Macro-fluidic (primary)	Macro-fluidic (primary)	Micro-biochemical (secondary)
Physical action	Forced, sustained blockage of the shared craniocervical outflow bottleneck, elevating venous pressure throughout the intracranial dural sinus system while arterial inflow continues; rapid release generates a discharge surge that may secondarily propagate into the continuous spinal venous network	CSF pressure gradient generated as the dural sac is tensioned and released, acting directly on the CSF-filled perivascular/interstitial compartment and, separately, coupling via Monro-Kellie to a corresponding venous pressure gradient wherever the CSF gradient reaches	Capillary/venule wall strain and possible rapid hypoxic onset during venous occlusion (mechanotransductive; hypoxic contribution untested, not excluded)
Primary target	Removal of dislodged, suspended protein via accelerated whole-brain venous-side efflux (occlusion elevates pressure and loosens adhesion; rebound performs the flushing across the shared outflow bottleneck); secondary, smaller spinal venous/lymphatic clearance during the same rebound phase	Agitation and dislodgement of adherent misfolded TDP-43/SOD1 throughout the perivascular/interstitial CSF space via two complementary routes: direct CSF-side agitation within this compartment (primary; acts throughout the craniospinal axis), and a Monro-Kellie-coupled venous pressure gradient reaching the same compartment from the venous side (predominantly cranial). Cranially, removal is addressed jointly with venous occlusion-rebound; in the spinal cord, the direct CSF-side route performs both agitation and removal	Candidate HSF-1 induction across the neurovascular unit (endothelium, pericytes, or astrocytes, by the general precedent that mechanical/hemodynamic stress activates HSF-1 in vascular tissue; no specific transducer or cell type is proposed as more likely than another); and/or hypoxia-mediated; EAAT2/GLT-1 stabilization if astrocytic HSF-1/HSP70 response occurs
Anatomical reach	Whole brain reliably during occlusion, via the shared outflow bottleneck; spinal cord not directly blocked (segmental drainage bypasses via azygos/hemiazygos/lumbar routes), but rebound surge may secondarily extend into spinal venous drainage, smaller than the cranial effect and uncharacterized	Direct CSF-side route: throughout the craniospinal axis, including the thoracolumbar cord, since it depends only on perivascular CSF rather than cranial-vault rigidity; magnitude decay with distance from the craniocervical junction is plausible but uncharacterized. Coupled venous route: near-uniform across the cranial vault via rigid-vault pressure transmission; predominantly cranial, with attenuated spinal extension not excluded but expected to be smaller than the cranial effect. Both pathways unconfirmed pending direct measurement	Intracranial compartment only
Motor neuron pool protected	Cortical UMN and bulbar LMN reliably (whole-brain occlusion-rebound effect); spinal UMN tracts and spinal LMN pools only via the secondary, uncharacterized rebound surge	Cortical UMN and bulbar LMN via the coupled venous route; spinal UMN tracts and spinal LMN pools throughout the cord via the direct CSF-side route, though this route's magnitude and any distance-dependent attenuation remain uncharacterized	UMN and bulbar LMN, if biochemical response occurs (unconfirmed)
HSF-1/HIF-1α dependence	Independent	Independent	HSF-1- and/or HIF-1α-dependent (proposed, non-exclusive); HIF-1α co-activation kinetics below 2 minutes uncharacterized, treated as open rather than excluded
Preclinical/physiological evidence	Rests on established venous-compression physiology (the same principle underlying the Queckenstedt test and internal jugular compression); indirect support from craniocervical venous anatomy for the whole-brain occlusion effect; spinal extension is not supported beyond anatomical plausibility of network continuity; neither phase directly tested	Indirect support from arterial-pulse/passive-venous-drainage asymmetry and from clinical treatment of intracranial pressure as approximately uniform in the rigid cranial vault; the Monro-Kellie CSF-to-venous coupling itself is well established (as for occlusion-rebound), but ACT-specific pressure magnitudes and the coupling's actual reach have not been directly measured. Spinal perivascular/paravascular fluid pathways, structurally analogous to the cranial glymphatic system, have been demonstrated anatomically [38,39], supporting the plausibility of the direct CSF-side route's spinal reach, though this evidence remains preliminary compared with cranial glymphatic characterization	Mechanical stress alone activates HSF-1/HSP70 in vascular tissue independent of ischemia, in cultured smooth muscle [24] and in vivo under elevated blood pressure [43]; extension to cerebral endothelium, pericytes, or astrocytes, and the specific transducer involved, remain untested. Chaperone-mediated EAAT2/GLT-1 stabilization is proposed but not demonstrated; motor neurons are separately documented to have a high threshold for HSF-1 activation, motivating interest in a non-neuronal source [44]
Human trial status	Not directly tested	Not directly tested	Not directly tested; two pharmacological trials targeting overlapping downstream biology did not improve outcomes in unselected ALS (ceftriaxone: EAAT2 upregulation [45]; arimoclomol: HSF-1 co-induction [46]), though a smaller SOD1-restricted arimoclomol trial showed a statistically inconclusive but favorable trend [47], plausibly reflecting a CHIP-mediated route [48]. Both used systemic delivery rather than CVPC's proposed local mechanism, so this negative result motivates rather than undermines the alternative, and neither trial's outcome bears directly on CVPC's own status

Synergy at the neurovascular unit

The macro-fluidic and micro-biochemical mechanisms proposed above are hypothesized to unfold on a biphasic timeline, adapted from the companion Alzheimer's disease framework [23] and refined here to reflect the additional venous mechanism developed in this review.

A Synchronous Phase (the immediate mechanical event, occurring within seconds) is hypothesized to encompass the full mechanical event: suboccipital venous occlusion-rebound and dural pull-recoil occurring concurrently within the same several-second ACT maneuver, rather than as separable or sequential techniques. Cranially, the two mechanisms converge on the same venous-side target through distinct pathways, as detailed above; in the spinal cord, where occlusion-rebound's reach is limited to a secondary, uncharacterized rebound effect, dural pull-recoil's direct CSF-side pathway is proposed as the primary mechanism of both agitation and removal.

A Metachronous Phase (the delayed biochemical response), corresponding to the micro-biochemical axis, is hypothesized to occur hours later, if astrocytic HSF-1 activation and resulting HSP70-mediated EAAT2 stabilization occur as proposed. Should this response occur, it would contribute incremental protection, plausibly concentrated in cortical UMN and bulbar LMN pools given the micro-biochemical axis's intracranial-only proposed reach (Table 1), on top of the Synchronous Phase's broader effect; it is not required to explain any protective effect ACT may produce, and the framework's central claim does not depend on it occurring.

Established evidence versus proposed framework

The evidentiary basis for this framework is uneven by design and should be read accordingly. The Monro-Kellie doctrine and the Queckenstedt test's coupling of venous compression to CSF pressure are established physiology, directly confirmed by phase-contrast MRI. The anatomical continuity of the craniocervical venous network and the arterial-pulsation-driven asymmetry of glymphatic influx versus efflux are likewise supported by direct evidence. What is proposed rather than established is the application of this physiology to a specific mechanical maneuver (ACT), what pressure magnitudes and timing this maneuver actually produces, and whether its proposed effects on glymphatic clearance and HSF-1 induction materialize as hypothesized, and whether any such effect is sufficient to slow disease propagation in ALS specifically. No component of this framework, macro-fluidic or micro-biochemical, has been directly measured in the context of ACT; every claim connecting established physiology to ACT's proposed effect in ALS is therefore inferential rather than demonstrated, and is presented as such throughout this review.

Limitations

First, this is a narrative rather than systematic review, appropriate to a hypothesis-generating framework spanning CSF/venous physiology, glymphatic-lymphatic clearance, and ALS-specific proteinopathy, but this format does not guarantee exhaustive coverage of the underlying literature in the way a systematic review with predefined search parameters would; the search strategy is reported in the "Literature search methodology" section for transparency, but selection of sources beyond the initial search, particularly foundational physiology papers identified through reference-list snowballing, involved judgment that a systematic protocol would have constrained.

Second, a related but distinct uncertainty concerns prion-like templating itself rather than its extracellular compartment. As noted in the Introduction, the evidence that TDP-43 and SOD1 template misfolding in native protein is substantially derived from cell culture, organoid, and animal-model systems; the extent to which this templating capacity, as opposed to other candidate mechanisms such as regional network vulnerability or spread of a non-templating diffusible stressor, actually drives clinical progression in human ALS remains incompletely established. This framework's central rationale depends on both propositions holding: that templated propagation is a clinically meaningful driver of human disease progression, and that a sufficient fraction of this propagation transits an extracellular or perivascular compartment accessible to glymphatic-lymphatic clearance. Should either proposition prove false or substantially overstated, the macro-fluidic axis's disease-modifying potential would be correspondingly reduced, independent of whether ACT achieves its proposed mechanical clearance effect.

Third, this framework's macro-fluidic axis presumes that a mechanically meaningful fraction of TDP-43/SOD1 propagation transits an extracellular or perivascular compartment accessible to glymphatic-lymphatic clearance. Prion-like propagation of both proteins is well established [3,4]. Still, propagation can also occur through routes that this framework does not address, including direct cytoplasmic transfer via tunneling nanotubes, closely apposed contact-dependent transfer, and exosome-mediated transfer, in which misfolded protein packaged within extracellular vesicles may be less accessible to bulk fluid clearance than freely suspended protein. The relative contribution of each route, including this vesicle-bound pathway, to the overall spread of disease in human ALS has not been quantified. Should extracellular/perivascular transit of freely suspended protein prove to be a minor contributor to propagation compared with these contact-dependent or vesicle-mediated mechanisms, the macro-fluidic axis's disease-modifying potential would be correspondingly reduced, regardless of whether ACT achieves its proposed clearance effect. This uncertainty is distinct from, and additional to, the uncertainty already noted above regarding whether ACT's proposed mechanical effects on CSF/venous dynamics occur as hypothesized.

Fourth, and more fundamentally, no component of ACT's proposed mechanism has been directly measured. The maneuver's actual intracranial and CSF pressure magnitudes, the timing relationship between occlusion onset and dural elongation within a single cycle, and whether either produces detectable glymphatic clearance change are all unknown quantities rather than established parameters; the 60%-70% body-weight thresholds cited from prior technical work [30] are load parameters applied to the maneuver, not measured physiological outputs of it.

Fifth, the framework's spinal-cord extrapolation rests on cranial glymphatic evidence and separately demonstrated spinal perivascular anatomy, rather than on direct spinal glymphatic measurement; DTI-ALPS evidence in ALS to date is itself cerebral, early-stage, and limited to a small number of cohorts. This reliance on downstream, clearance-consistent proxies rather than direct measurement of the disease-defining misfolded species is not unique to this paper; the companion Alzheimer's disease framework [23], from which this paper's micro-biochemical axis is drawn, proposes an analogous validation pathway using DTI-ALPS and peripheral blood exosomal HSP70 quantification rather than direct visualization of amyloid-beta/tau clearance or intracellular tau stabilization, indicating that this indirection is a structural feature of the series' validation strategy rather than a limitation specific to ALS.

Sixth, the micro-biochemical axis introduces additional uncertainty beyond the macro-fluidic axis. The wall-distension-to-HSF-1 pathway has been demonstrated in arterial smooth muscle but not in venous cerebral endothelium, pericytes, or astrocytes, and no specific molecular transducer for this response in the cerebral neurovascular unit has been identified; and the kinetics of HIF-1α induction under sub-2-minute venous occlusion remain uncharacterized in either direction. The two prior pharmacological trials discussed above, ceftriaxone and arimoclomol, targeted overlapping downstream biology but used systemic delivery; their null or inconclusive results neither support nor undermine CVPC's proposed local, non-pharmacological mechanism, and no data currently exist that directly address CVPC's own status.

Seventh, no non-invasive method directly confirms occlusion of the suboccipital venous plexus in vivo. The plexus is a diffuse, valveless venous confluence around the craniocervical junction rather than a single vessel with an established Doppler or duplex protocol. In contrast, adjacent vessels do not provide an interpretable substitute: the vertebral vein is structurally shielded within the bony transverse foramina and carries pooled drainage from the vertebral venous plexus along the entire cervical spine rather than a dedicated outflow specific to the suboccipital confluence, while the internal jugular vein is anatomically distinct from the plexus and already near-collapsed at baseline in erect posture. Any proposed validation pathway, therefore, lacks a direct physiological manipulation check to confirm that the mechanical event occurred as intended and relies on DTI-ALPS as a downstream proxy without independent confirmation at the mechanism's proposed site of action. Prior work has proposed the onset of mild presyncope itself as a real-time perceptual signal that suboccipital occlusion has reached functional threshold, with its absence indicating inadequate occlusion rather than mechanism failure [27,30]; this signal has not been instrumented or independently validated against direct pressure measurement, but offers a candidate proxy at the mechanism's site of action beyond DTI-ALPS alone.

Eighth, this framework does not address the magnitude or durability of the clearance effect produced by either a single session or sustained daily practice, particularly in relation to the continuous and cumulative nature of prion-like templating. This question can only be resolved empirically and is the explicit target of the staged validation pathway described below.

Oncologic safety

Because the macro-fluidic axis is this framework's primary mechanism, its physical action carries the more immediate oncologic safety consideration. Both suboccipital venous occlusion-rebound and dural pull-recoil, delivered together in the same ACT maneuver, apply mechanical force, pressure changes, and accelerated fluid/venous drainage directly to the craniocervical junction and spinal cord, including the thoracolumbar segments reached via dural pull-recoil's direct CSF-side pathway. In a patient with an undiagnosed or known mass lesion at this site, this loading could plausibly pose an independent structural risk, from local tissue irritation to mechanical disruption of a fragile or vascular lesion, regardless of whether the micro-biochemical axis is active.

A secondary consideration applies only if the proposed HSF-1/HSP70 response occurs: HSP70 is protective against misfolding in degenerating tissue but can also support tumor cell survival under proteotoxic stress [49]. Because any such induction is proposed to remain localized to the intracranial compartment rather than exported systemically as in RIPC [50], the theoretical concern in patients with undiagnosed intracranial malignancy would be limited to that compartment rather than eliminated.

Taken together, ACT should be considered contraindicated or used with caution in patients with a known or suspected primary neoplasm anywhere within the CNS that is reached by this framework's proposed mechanisms, including the brain, craniocervical junction, and spinal cord, and is intended for clinician-supervised application only. This framework is theoretical and does not constitute clinical practice guidance.

Methodological framework and priorities for clinical validation

The staged design below is organized around the same three-level distinction drawn in the "Established evidence versus proposed framework" section: whether ACT produces a measurable mechanical/physiological perturbation, whether that perturbation accelerates clearance, and whether any clearance effect is sufficient to alter clinical progression. Tier 1 and Tier 1a address the first two levels; Tier 2's plasma neurofilament light chain (NfL) outcome, introduced below as a "clearance-independent" measure, addresses the third separately, and neither level should be read as confirming the others.

An animal model is not proposed as a preliminary step. ACT is a posture correction and spinal decompression maneuver [27] in the same category as manual therapy and axial spinal traction, interventions that have a long history of proceeding directly to human study without a preceding animal model, since the intervention carries no systemic pharmacological exposure to characterize and depends on active participant engagement (ACT-SA) or practitioner-operator-driven delivery (ACT-OD) [30] that a non-human model cannot meaningfully replicate. On this basis, proceeding directly to a monitored human study with directly observable physiological readouts is proposed as the more appropriate first step than a rodent or other animal proxy. 

This framework adopts the Tier 1/Tier 2 staged design established for ACT generally [27]. Tier 1 is a combined safety and tolerability study: continuous monitoring of blood pressure, heart rate, pulse oximetry, and respiratory rate during a single supervised ACT session in healthy volunteers. This vital-sign panel is intended to capture the clinical safety endpoint that matters most at this stage, namely whether the maneuver produces a hemodynamic response, presyncope, bradycardia, or hypotension, severe or prolonged enough to warrant halting or modifying the protocol, rather than to serve as a direct measurement of the intracranial, venous, or CSF pressure changes ACT is hypothesized to produce. As discussed in the "Limitations" section, no non-invasive method currently exists to confirm suboccipital venous plexus occlusion in vivo, so instrumentation intended to directly characterize the proposed mechanism, rather than to monitor for clinical safety signals, would not close this gap at the present stage; Tier 1 is accordingly scoped to the latter. Tier 1 is disease-nonspecific by design and is not proposed separately in ALS patients as a first step; a result showing no clinically significant adverse vital-sign perturbation, obtained in any population, is proposed to support proceeding to ALS-specific testing, without requiring an independent repetition of Tier 1 in healthy volunteers for this disease.

The direction of any autonomic response to suboccipital occlusion-rebound cannot be assumed in advance. Bradycardia and hypotension are cited above as illustrative concerns, but because the maneuver is a brief, Valsalva-like load against a congested rather than ischemic venous bed, tachycardia and hypertension are at least as plausible a response, particularly during the occlusion phase before rebound. Tier 1 monitoring is accordingly designed to be sensitive to autonomic change in either direction rather than to a single predicted pattern. Stopping criteria are fixed prospectively as heart rate and blood pressure thresholds set symmetrically around baseline, so that a clinically significant deviation in either direction halts or modifies the session. A mandatory recovery interval between cycles requires vital signs to return to baseline before the next cycle proceeds. Rather than excluding candidates with these conditions outright, the protocol instead names known autonomic dysfunction, arrhythmia history, and uncontrolled hypertension explicitly as requiring supervising-practitioner screening and individualized risk assessment before enrollment, shifting the burden of judgment onto documented practitioner evaluation rather than leaving it implicit within a general category of cardiovascular instability. Consistent with prior protocol design, presyncope onset within the target hold duration is treated as an indicator of adequate occlusive load, but its absence is treated as a signal to pause and reassess rather than as grounds to increase applied force or hold duration; escalation of either parameter beyond the fixed values specified below is not proposed under any circumstance.

Within this same healthy-volunteer stage, a brief acute sub-study, Tier 1a, is proposed: a within-session pre- and post-maneuver DTI-ALPS comparison using a small, fixed number of ACT-OD-delivered cycles within established tolerance, to test feasibility before committing to the longer Tier 2 protocol. ACT-OD is used here for operational consistency across participants, since standardized cycle timing is easier to reproduce with a practitioner-operated device, and some participants are unable to relax sufficiently during self-administered ACT [30]. This reflects a choice about delivery consistency in the research setting rather than a mechanistic distinction between modes, which this framework treats as equivalent. No prior study has tested DTI-ALPS sensitivity to a brief mechanical perturbation of this kind, so Tier 1a is treated as a first, exploratory test of feasibility rather than as a dose calibrated against any established detection threshold; a null result is uninformative as to mechanism and should not be read as evidence against the macro-fluidic axis. Consistent with prior protocol design [27], candidates who reliably achieve presyncopal onset within the target hold duration are considered to have received adequate tensile and occlusive load; absence of presyncope during a given cycle may indicate insufficient occlusion rather than a failure of the underlying mechanism, a distinction relevant to interpreting a null Tier 1a result.

Given the possibility of disease-related alteration to perivascular architecture or CSF dynamics in ALS that a healthy-volunteer cohort would not capture, disease-specific Tier 1 reconfirmation is proposed before proceeding to Tier 2. This is also standard clinical research practice independent of that physiological rationale: even once Tier 1 has passed in healthy volunteers, each ALS participant would still undergo their own supervised, monitored first exposure to the maneuver before enrollment in Tier 2, rather than being enrolled directly on the strength of another population's data. This reconfirmation is staged by ambulatory capacity: ambulatory patients able to self-administer ACT-SA or receive ACT-OD first, with the same vital-sign monitoring repeated within the ALS population itself, before extending to non-ambulatory patients using ACT-OD, the only mode available to this group.

Two limitations qualify any proposed direct physiological confirmation stage. First, the suboccipital venous plexus, comprising the vertebral venous plexus and posterior emissary channels, is anatomically enclosed by the transverse foramina and the posterior arch of the atlas [36], structures that limit ultrasound access to this plexus specifically; duplex or transcranial Doppler can characterize internal jugular vein flow as a downstream, indirect correlate of overall craniocervical venous dynamics, but cannot directly insonate the suboccipital plexus itself, and a jugular-flow signal cannot be attributed uniquely to suboccipital occlusion as opposed to other concurrent hemodynamic changes. Second, and more fundamentally, ACT delivers suboccipital venous occlusion-rebound and dural pull-recoil concurrently within a single maneuver by design; any downstream physiological or biomarker change observed following ACT, whether DTI-ALPS change or NfL change, cannot be mechanistically attributed to one component rather than the other without an experiment that delivers each mechanism in isolation, which would depart from ACT as proposed and practiced. This framework accordingly does not propose to discriminate the relative contribution of suboccipital venous occlusion-rebound versus dural pull-recoil at the validation stages described below; the macro-fluidic axis is proposed and tested as a single, bundled mechanism, and component-isolation studies, for example, occlusion delivered without traction or traction delivered without occlusion, are noted here as a possible but separate line of future investigation rather than a required element of the staged validation pathway.

Tier 2, comprising sustained daily practice over 3-6 months with repeat DTI-ALPS measurements relative to baseline, is proposed for this ALS-confirmed cohort. Because Tier 2 tests the cumulative effect over months rather than a single-session acute signal, it does not depend on the delivery precision that Tier 1a requires and, accordingly, permits either ACT-SA or ACT-OD, depending on what a given patient can access and sustain, consistent with the ambulatory-capacity staging above. Plasma NfL, an already validated ALS progression biomarker [13], would be introduced at this stage as a longer-term, clearance-independent outcome measure to confirm durable disease modification, distinct from the acute Tier 1/Tier 1a safety and feasibility readouts.

Because ACT, as described here, has never been directly tested, the parameters specified for Tier 1a and Tier 2 are fixed rather than left open to iterative adjustment to avoid rendering the hypothesis unfalsifiable in practice. The applied load for ACT-OD is fixed at the full lift-off of the participant from the seated position, corresponding to the established 60%-70% body-weight range [30]. Hold duration is fixed at approximately 5 seconds, extending to a maximum of 10 seconds only if presyncopal onset remains tolerable; 10 seconds is treated as an absolute ceiling not to be exceeded regardless of symptom status. Session frequency is fixed at 3-5 times daily, informed by the author's own sustained personal practice at this frequency without adverse effect over several years; this exceeds the more conservative 1-to-3-times-daily frequency specified in prior technical reports in this series [26,27,30], reflecting the view that a disease with ALS's prognosis and absence of disease-modifying options warrants a correspondingly less conservative dosing baseline than a preventive or maintenance application, though this frequency remains unstudied at the population level and is not established to be safe or superior to the lower range. This figure is accordingly presented as a hypothesis to be tested by Tier 1 safety monitoring, not as a validated or recommended dose; Tier 1's stopping criteria, rather than the author's personal practice, are what would determine whether 3-5 times daily is tolerable in a monitored population. Under these fixed parameters, a positive Tier 2 result requires favorable change on both outcome measures: DTI-ALPS stabilization or elevation relative to baseline, since no existing longitudinal ALS cohort has established a reliable quantitative decline rate against which a smaller, attenuated decline could be distinguished from measurement noise, and a plasma NfL trajectory over the same 3-6 months, attenuated relative to published untreated natural-history rates. A decline in DTI-ALPS relative to baseline, a plasma NfL rise consistent with or exceeding published natural-history rates, or both, is sufficient to falsify the macro-fluidic axis's Tier 2 claim, since either measure alone, when declining, is treated as falsifying rather than only their joint decline. This dual-endpoint requirement is deliberately stricter than the single-surrogate standard that supported tofersen's own accelerated approval [13], reflecting the view that a mechanically bundled, non-pharmacological intervention with no target-engagement confirmation of its own should be held to a higher evidentiary bar before any efficacy claim is entertained. This is intended as a concrete, prospectively fixed stopping rule rather than an open-ended license to iterate dosing until a positive result is obtained.

## Conclusions

ALS is characterized by prion-like propagation of misfolded TDP-43 and SOD1 across motor neuron pools. This framework proposes ACT, delivering suboccipital venous occlusion-rebound and dural pull-recoil concurrently, as a mechanically grounded route to disease modification in ALS: interrupting prion-like propagation by clearing suspended misfolded TDP-43 and SOD1 before they template neighboring cells, without requiring any biochemical response from the recipient cell. A secondary, mechanistically distinct micro-biochemical axis, examining whether the same occlusion produces sufficient wall strain to activate HSF-1 across the neurovascular unit and thereby stabilize astrocytic EAAT2 against the glutamate excitotoxicity described in the Introduction, targets a downstream consequence of proteotoxic stress rather than propagation itself; it is not required by, and does not displace, this primary claim.

This framework is entirely theoretical at present: neither pressure mechanism has been directly measured, and no clinical data yet exist for ACT as described here. Two pharmacological trials targeting the framework's secondary biology, ceftriaxone and arimoclomol, tested systemic delivery, not CVPC's proposed local, non-pharmacological paradigm, so their null or inconclusive results motivate this alternative rather than bearing on CVPC's own status. This distinction, mechanical clearance versus pharmacological induction, is the framework's central departure from prior ALS therapeutic strategies. Extension to other proteinopathy-driven neurodegenerative diseases, including Parkinson's disease, Huntington's disease, spinocerebellar ataxia, and fatal familial insomnia, is contingent on the physiological mechanisms proposed here first being validated in ALS; forthcoming companion papers addressing these diseases propose the same mechanisms as an analogous hypothesis in each disease context, not as an extension of a demonstrated effect, and each requires its own independent confirmation. No therapeutic efficacy claim, in ALS or elsewhere, is made or implied prior to that validation. ACT in ALS remains a falsifiable hypothesis awaiting empirical test, with the Tier 2 validation pathway requiring concordant improvement in both DTI-ALPS and plasma NfL to support the framework's central claim, and decline or failure to improve in either endpoint alone sufficient to falsify it. ACT is a supplementary adjunct under investigation, not a validated intervention or a replacement for standard care.
